# Releasing the Bubbles: Nanotopographical Electrocatalyst Design for Efficient Photoelectrochemical Hydrogen Production in Microgravity Environment

**DOI:** 10.1002/advs.202105380

**Published:** 2022-01-21

**Authors:** Ömer Akay, Jeffrey Poon, Craig Robertson, Fatwa Firdaus Abdi, Beatriz Roldan Cuenya, Michael Giersig, Katharina Brinkert

**Affiliations:** ^1^ Department of Physics Free University Berlin Arnimallee 14 14195 Berlin Germany; ^2^ Department of Interface Science Fritz Haber Institute of the Max Planck Society 14195 Berlin Germany; ^3^ Department of Chemistry University of Warwick Gibbet Hill Road Coventry CV4 7AL UK; ^4^ Institute for Solar Fuels Helmholtz‐Zentrum Berlin für Materialien und Energie GmbH Hahn‐Meitner‐Platz‐1 14109 Berlin Germany; ^5^ Institute of Fundamental Technological Research Polish Academy of Science Pawińskiego St. 5b Warsaw 02‐106 Poland

**Keywords:** electrocatalyst nanotopography, hydrogen evolution, microgravity, photoelectrocatalysis, (photo‐)electrochemical gas bubble evolution, shadow nanosphere lithography

## Abstract

Photoelectrochemical devices integrate the processes of light absorption, charge separation, and catalysis for chemical synthesis. The monolithic design is interesting for space applications, where weight and volume constraints predominate. Hindered gas bubble desorption and the lack of macroconvection processes in reduced gravitation, however, limit its application in space. Physico‐chemical modifications of the electrode surface are required to induce gas bubble desorption and ensure continuous device operation. A detailed investigation of the electrocatalyst nanostructure design for light‐assisted hydrogen production in microgravity environment is described. p‐InP coated with a rhodium (Rh) electrocatalyst layer fabricated by shadow nanosphere lithography is used as a model device. Rh is deposited via physical vapor deposition (PVD) or photoelectrodeposition through a mask of polystyrene (PS) particles. It is observed that the PS sphere size and electrocatalyst deposition technique alter the electrode surface wettability significantly, controlling hydrogen gas bubble detachment and photocurrent–voltage characteristics. The highest, most stable current density of 37.8 mA cm^−2^ is achieved by depositing Rh via PVD through 784 nm sized PS particles. The increased hydrophilicity of the photoelectrode results in small gas bubble contact angles and weak frictional forces at the solid–gas interface which cause enhanced gas bubble detachment and enhanced device efficiency.

## Graphical abstract

Rhodium electrocatalyst nanotopographies fabricated by shadow nanosphere lithography on p‐InP for photoelectrochemical hydrogen production in microgravity environment are investigated. The results reveal that this lithography technique combined with physical vapor deposition results in the formation of a nanostructured, hydrophilic Rh layer which leads to enhanced hydrogen gas bubble desorption, as well as high and stable photocurrent densities in reduced gravity.

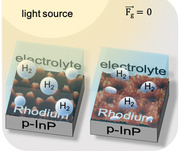

## 1 Introduction

Long‐term space missions face similar challenges to the realization of a sustainable energy economy on earth: renewable energy systems are required which convert and store energy in the form of fuels, electricity, and chemicals for day and night operation at high efficiency, stability, and durability. Hydrogen (H_2_) is a key player in this scenario for both, terrestrial and space applications, due to its high energy density (143.0 MJ kg^−1^).^[^ ^1^ ^]^ Another advantage is that hydrogen can be directly used in a Sabatier reactor to convert carbon dioxide to water and methane which is currently already used in the life support system on the International Space Station (ISS) for carbon dioxide removal.^[^ ^2^, ^3^ ^]^ In the past decades, artificial photosynthesis systems have been largely developed and explored for solar hydrogen production. Integrated, technologically advanced III–IV semiconductor‐electrocatalyst systems currently realize solar water‐splitting and simultaneous hydrogen production at the highest technologically possible efficiency and long‐term stability.^[^ ^4^, ^5^, ^6^, ^7^, ^8^ ^]^ Their investigation for space applications is therefore evident as they could potentially be used for solar‐to‐chemical energy conversion reactions and thereby complementing currently existing life support technologies. The absence of buoyancy and the resulting absence of macroconvectional processes, however, significantly challenge the application of (photo‐)electrochemical systems in reduced gravitational environments.^[^ ^9^, ^10^, ^11^, ^12^, ^13^, ^14^ ^]^ As reported earlier in water electrolysis studies in microgravity, the absence of density‐driven phase separation causes hindered desorption of electrochemically produced gas bubbles from the electrode surface, leading to the formation of gas bubble froth layers and bubble coalescence.^[^ ^15^, ^16^, ^17^, ^18^ ^]^ This results in turn in an increased ohmic resistance in proximity to the electrode surface, ultimately leading to a significant increase in the reactions’ overpotential^[^ ^19^ ^]^ and possibly the loss of potentiostatic control. Rotational devices have been developed to introduce phase separation, e.g., on the ISS, which however also increase the energy bill: approximately 1.5 kW out of the 4.6 kW used by the entire Environmental Control and Life Support System (ECLSS) on the ISS is consumed by the Oxygen Generator Assembly (OGA), oxidizing water into O_2_ and H_2_.^[^ ^20^ ^]^ The high energy demand results from the electrochemical potential for the reaction (+1.23 V *vs* RHE) and the present ohmic, concentration, and activation overpotentials significantly increase the required energy input. A prominent terrestrial example of how gas bubbles significantly influence the electrochemical cell characteristics is the industrial chlor‐alkali electrolysis, where ohmic polarization losses at the anode due to chlorine gas production lead to high reaction voltages.^[^ ^21^ ^]^ Recently, gas bubble formation has also become the focus of several photoelectrochemical device studies for terrestrial applications. Decelerated gas bubble desorption from the electrode surface causes light reflection from bubbles adhering to the electrode surface,^[^ ^22^, ^23^, ^24^ ^]^ the blockage of catalytically active sites,^[^ ^22^, ^24^ ^]^ and limited substrate and product transfer to and from the electrode surface.^[^ ^24^ ^]^ These are all factors which significantly lower the overall device output. Effective gas bubble desorption is thus a key element to consider in the design of efficient (photo‐)electrodes also for terrestrial applications.

Recently, we have investigated photoelectrochemical hydrogen production at the Bremen Drop Tower, ZARM (Center of Applied Space Technology and Microgravity, Germany), where microgravity (10^−6^ *g*) is generated for 9.2 s during free fall. We used shadow nanosphere lithography (SNL) to introduce an electrocatalyst nanotopography directly on the semiconductor surface for catalytic ‘hot‐spot’ formation.^[^ ^17^, ^25^, ^26^, ^27^, ^28^, ^29^ ^]^ The studied model system, p‐indium phosphide coated with the electrocatalyst rhodium, could efficiently produce hydrogen upon illumination (70 mW cm^−2^, W‐I lamp) at current densities >15 mA cm^−2^ for the duration of free fall.^[^ ^17^, ^18^ ^]^ Here, we investigate the electrocatalyst nanotopographies fabricated by SNL in reduced gravitation in more detail and use physical vapor deposition (PVD) and photoelectrodeposition (PED) to deposit Rh through a mask of a hexagonally closed‐packed monolayer of monodispersed polystyrene (PS) spheres. Our results reveal that by altering the PS sphere size between 252 and 784 nm as well as the Rh deposition method, the hydrogen gas bubble desorption process can be judiciously controlled in microgravity environment which results in significantly optimized photoelectrochemical (PEC) half‐cell characteristics. Continuous gas bubble release was observed even at high current densities up to 34 mA cm^−2^ in the absence of buoyancy. The investigated electrode surface design could provide a step‐change in the utilization of (photo‐)electrode systems for space applications, and it could also improve the energy efficiency of currently existing (photo‐)electrolytic terrestrial devices.

## 2 Results

Photoelectrochemical hydrogen production in reduced gravitational environments (10^−6^ *g*) was realized at the Bremen Drop Tower.^[^ ^30^ ^]^ The capsule, containing the experimental set‐up and supporting equipment, was accelerated in 0.25 s to a speed of 168 km h^−1^ and flew up to the top of the 120 m tall drop tube before falling down into a deceleration chamber after 9.2 s. A drop sequence was programmed prior to capsule launch to automatically collect the photoelectrochemical data sets.

### 2.1 Photoelectrochemical Characteristics

**Figure 1:**
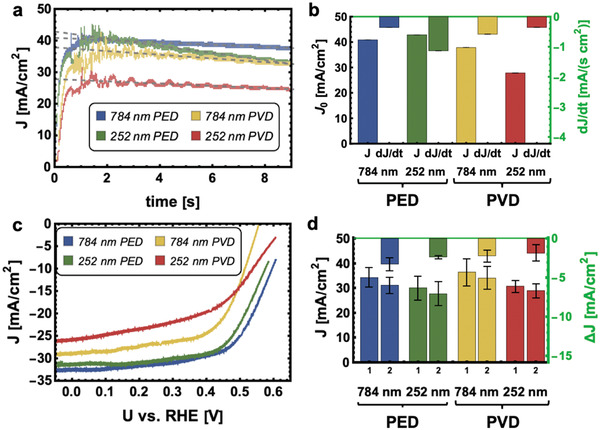
a) Chronoamperometric measurements of p‐InP photoelectrodes with nanostructured Rh electrocatalyst layers carried out for 9.2 s in microgravity environment at an applied potential of −0.09 V *vs* RHE. A linear regression was calculated for each Rh electrocatalyst morphology. b) Data determined by linear regression are represented in double‐*y* bar charts. The initial current density, *J*_0_, and the loss of *J* in the course of the experiment are indicated by the negative slope d*J*/dt for each sample. c) Second reduction cycle out of three complete cycles recorded in microgravity environment during 9.2 s. The scan rate was 326 mV s^−1^. d) The mean values for maximum current densities of the first two cycles and the difference of both were determined statistically. The difference in the current density drop between the first two cycles is shown as a negative value. The error bars represent standard deviations from three independent photoelectrochemical measurements during free fall with new electrodes for each measurement. All measurements were carried out in an electrolyte containing 1 m HClO_4_(aq) with the addition of 1% (v/v) 2‐propanol and a light intensity of 100 mW cm^−2^ (W‐I lamp).

Surface modifications of the p‐InP photoelectrodes prior to electrocatalyst deposition were carried out as previously described.^[^ ^17^, ^18^ ^]^ PS spheres with the sizes 252 or 784 nm, respectively, were directly deposited onto the p‐InP surface. Rh was deposited through the PS sphere mask by using either PED or PVD.^[^ ^6^, ^31^, ^32^ ^]^ The PS particles were removed prior to the test using toluene and subsequent Ar plasma treatment. The resulting set of four electrodes with at least three identical control samples each was tested in drop tower experiments. Before capsule release, the electrodes were immersed in an electrolyte of 1 m HClO_4_(aq) with the addition of 1% (v/v) isopropanol to lower the surface tension.^[^ ^33^ ^]^ Results from chronoamperometric (CA) and cyclovoltammetric (CV) experiments at 100 mW cm^−2^ illumination (W‐I lamp) during free fall are shown in **Figure** **1**. The control samples were used to determine the standard deviation in the photocurrent density measurements.

Despite that all four photoelectrodes perform well in microgravity environment, significant differences in the photoelectrochemical characteristics can be observed dependent on the fabricated electrocatalyst nanotopography. In chronoamperometric (CA) measurements, the PED photoelectrodes result in higher starting currents of up to 42.9 mA cm^−2^ (252 nm particle size), whereas the PVD samples show lower initial current densities of up to 37.84 mA cm^−2^ (784 nm particle size). The PVD sample made with 252 nm PS spheres shows the lowest initial current density of 27.8 mA cm^−2^. During the course of the experiment, the current densities decreased in all samples (Figure 1b). The steepest decrease of d*J*/dt = −1.1 mA cm^−2^ s^−1^ is observed for the 252 nm PED samples and the smaller decreases of d*J*/dt = −0.4 mA cm^−2^ s^−1^ are recorded for the 784 nm PED samples, whereas photoelectrodes made with PS sphere masks of 784 nm in diameter and Rh deposited via PVD result in a current density decrease of d*J*/dt = −0.6 mA cm^−2^ s^−1^. This observation is also reflected in the photocurrent–voltage (*J*–*V*) measurements. Figure 1c shows the second out of three recorded voltammetric cycles during the 9.2 s of free fall (scan rate: 326 mV s^−1^). The 252 nm PED sample shows very high short‐circuit current densities (*J*_sc_) of up to 42.6 mA cm^−2^, whereas the PED samples made with PS spheres of 784 nm diameter show a lower *J*_sc_ value of 40.9 mA cm^−2^ (Figure 1a,b). Both PVD samples also show lower *J*_sc_ values of 37.8 mA cm^−2^ (PS particle size 784 nm) and 27.8 mA cm^−2^ (PS particle size 252 nm), respectively. Comparing however the current density loss Δ*J* between the first and second reduction cycle in reduced gravitation, the PVD samples were subject to slightly lower losses of Δ*J* = 2.2 ± 0.8 mA cm^−2^ for the 784 nm and Δ*J* = 1.8 ± 1.1 mA cm^−2^ for the 252 nm PS particles, respectively. For the PED samples, Δ*J* values of 3.3 ± 0.8 mA cm^−2^ (784 nm PS particle size) and 2.4 ± 0.2 mA cm^−2^ (252 nm PS particle size), respectively, are calculated. The same trend can also be observed at a light intensity of 50 mW cm^−2^ (Figure S1, Supporting Information). Although the photocurrent density difference Δ*J* is more significant at 100 mW cm^−2^ illumination, it is evident that the 784 nm PED sample shows the highest current density drop between the first and second CV (Δ*J* = 3.3 ± 0.8 mA cm^−2^), whereas the PVD samples perform nearly identically well, producing short circuit current densities of up to 36.0 ± 5 mA cm^−2^. Despite the initially lower current densities, the PVD samples—particularly the 784 nm PS sphere size samples—produce nearly stable photocurrent densities in CV and CA measurements, giving them an advantage in application over PED samples.

### 2.2 Light Absorption and Rate Constants of Charge Transfer and Surface Recombination in Terrestrial and Microgravity Environments

All samples—except for the 252 nm PVD sample—give rise to higher photocurrent densities than previously reported with p‐InP photoelectrodes coated with an Rh electrocatalyst layer in terrestrial experiments, where photocurrent densities of up to 35 mA cm^−2^ have been observed under W‐I illumination (105 mW cm^−2^).^[^ ^33^, ^34^ ^]^ To investigate the impact of the Rh nanostructure on the *J–V* characteristics, incident photon‐to‐current efficiency (IPCE), intensity‐modulated photovoltage spectroscopy (IMVS), and intensity‐modulated photocurrent spectroscopy (IMPS) measurements were carried out terrestrially. All IPCE measurements were normalized with respect to the 784 nm PVD sample which generates the highest photocurrent density during both, W‐I (100 mW cm^−2^) and Xe lamp (AM 1.5 G) illumination, to minimize absorption differences in terrestrial and microgravity environments due to external parameters influencing the light absorption characteristics such as the gas bubble evolution behavior. As evident from Figure S2a,b (Supporting Information), the highest photocurrent densities per wavelength are recorded with the 784 nm PVD and the 252 nm PED samples, whereas the 252nm PVD and the 784 nm PED samples show a nearly identical absorption behavior during illumination with a W‐I lamp (100 mW cm^−2^) and a Xe lamp (AM 1.5 G). The normalized integrated photocurrent densities show the same trend (Figure S2c,d, Supporting Information). Here, the integrated photocurrent density of the 784 nm PED photoelectrode is about 16.5% lower than the one observed with the 784 nm PVD sample when a W‐I lamp was used for illumination, which is even lower (17.5%) when the sample was irradiated using the Xe lamp. The same observation was made for the 252 nm PVD sample, which generally shows a higher photocurrent density than the 784 nm PED sample, but still a significant lower value for the W‐I lamp (13.7%) and the Xe lamp (14.9%) in comparison to the 784 nm PVD sample. The 252 nm PED sample shows nearly an identical integrated photocurrent density compared to the 784 nm PVD sample. Although the overall absorption properties of the photoelectrodes remain similar, the IMPS measurements reveal quite significant differences between the samples (Figure S3, Supporting Information): the highest charge recombination rate constants (*k*_rec_) is observed for the 252 nm PVD sample, followed by the 252 nm PED photoelectrode (Figure S3a, Supporting Information). The effective electron transfer rate (*k*_tr_) is on the contrary also highest for the 252 nm PED sample (Figure S3b, Supporting Information), followed by the 252 nm PVD photoelectrode. For the 784 nm PVD and 784 nm PED samples, the charge recombination rates are smaller, but *k*_tr_ is significantly higher in the 784 nm PVD samples than in the 784 nm PED ones. The high electron transfer rate of the 252 nm PED sample is reflected in the high photocurrent densities initially observed in the CA measurements in microgravity, whereas the high charge recombination rate shown by the 252 nm PVD sample certainly has a major impact on the low photocurrent densities observed in Figure 1a. The comparably low recombination rates and the higher electron transfer rates of the 784 nm PVD sample are certainly factors influencing the high and stable photocurrent densities observed in microgravity environment. IMVS measurements (Figure S3c, Supporting Information) furthermore reveal that the 784 nm PVD samples possess the longest electron lifetime,*τ*_n_ (267 µs), which is significantly different from the other nanostructured samples, where electron lifetimes of 33 µs (252 nm PVD) to 53 µs (784 nm PED) are observed (**Table** **1**). A longer electron lifetime could be a significant advantage for photoelectrodes in reduced gravitation, where gas bubble desorption processes are slower than in terrestrial environments, leading to a blockage of catalytically active sites for longer time periods. Here, longer electron lifetimes could potentially accommodate the slower gas bubble desorption process and thus allow a continuous reduction of reactants at the electrode surface. Based on the discussed photoelectrochemical properties it remains however difficult to explain the high photocurrent densities initially observed in nearly all CA measurements in microgravity. These are certainly influenced by the factors discussed above, although they only provide possible explanations for the different *J–V* characteristics.

**Table 1:** Electron lifetimes for the p‐InP‐Rh photoelectrodes calculated from terrestrial IMVS measurements in 1 m HClO_4_(aq) with the addition of 1% (v/v) 2‐propanol (see Nyquist plot in Figure S2c, Supporting Information). Electron lifetimes were calculated according to *τ*_n_ = (2*π f*_max_)^−1^

Sample	*f*_max_ [Hz]	*τ*_n_ [µs]
252 nm PVD	4763.96	33
784 nm PED	2982.48	53
252 nm PED	3769.41	42
784 nm PVD	596.36	267

External factors which are inherent to reduced gravitation have to be considered for data interpretation as well. Previously, we modeled the current–voltage behavior of a photoelectrochemical device in microgravity environment, by considering mass transport limitations in the reaction rate due to gas bubbles adhering to the electrode surface on p‐InP photoelectrodes coated with a continuous Rh thin film. One possibility, which has not been considered in optoelectronic models of PEC devices in terrestrial and microgravity environment, is gas bubble detachment from the electrode surface without the removal from the proximity of the electrode surface due to the absence of buoyancy. This effect is observed here (see video recordings during the 9.2 s of free fall). Despite not limiting the mass transfer of reactants due to their detachment from the electrode surface, the gas bubbles remain in close proximity and could thus have a mirroring and light‐concentrating effect of the incident light. This hypothesis was modeled based on an established attempt to account for a nonuniform transmission of light to the photodiode using a transmission factor *f*_T_, which was introduced by Fountaine et al. to account for light absorption and reflection by the electrocatalyst layer in integrated semiconductor‐electrocatalyst devices.^[^ ^35^ ^]^ The introduction of a transmission factor accounting for increased local light intensities due to light reflections at the gas bubble–electrolyte interface (*f*_T,r_) leads to an analytic equation for the current–voltage behavior of a nanostructured coupled electrocatalyst–semiconductor device in which *k* is the Boltzmann constant, *T* (K) is the temperature which is assumed to be 298.15 K, *q* is the elementary charge, *j*_0_ is the dark current, *j*_L_ is the light‐limited current of the photodiode, *R* is the universal gas constant, *n*_e_ is the number of electrons associated with the reaction which is 2 in this case, *F* is the Faraday constant, *j*_0,cat_ is the catalyst exchange current density, *f*_SA_ is the catalyst surface area factor relative to the planar device area, and *f*_T,r_ is the transmission factor accounting for light reflection

(1) $$V_{\mathrm{PEC}}\;\left(j\right)=\frac{kT}{q}\;\ln\left(\frac{j_{L}f_{T,r}-j}{j_{0}}+1\right)-\frac{2RT}{n_{e}F}\sin h^{-1}\left(\frac{i}{2j_{0,\mathrm{cat}}f_{\mathrm{SA}}}\right)$$

**Figure 2:**
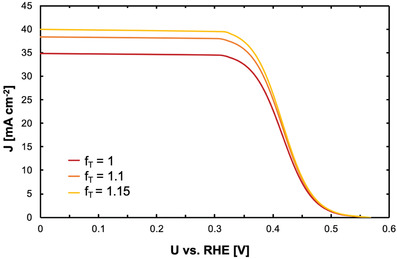
Simulations of the *J*–*V* characteristics of the 784 nm PED sample in reduced gravitational environments (see text for details). Illumination was assumed to occur at 100 mW cm^−2^ through a W‐I lamp. The electrolyte addition was 1 m HClO_4_(aq) with the addition of 1% (v/v) isopropanol. To simulate the effect of local light intensity increases on the light‐limited current of the photodiode due to gas bubbles in proximity to the electrode surface, the transmission factor, *f*_T,r_, was varied. The simulation shows that already small changes of the transmission factor have a significant impact on the photocurrent density.

If *f*_T,r_ is only slightly increased from 1 to 1.15, the overall photocurrent density increases significantly as shown in **Figure** **2**. This hypothesis provides one possible explanation for the higher photocurrent densities observed in CA measurements here and requires further investigation, e.g., by measuring the IPCE of photoelectrodes in microgravity environment. The results have to then be integrated into extended models of the interfacial processes occurring in integrated semiconductor‐electrocatalyst devices in reduced gravitational environments.

### 2.3 Surface Morphology

To elucidate the impact of the surface morphology, structure and composition on the photoelectrochemical characteristics of the PVD and PED nanostructured photoelectrodes in microgravity environment, the electrode surfaces were characterized by structural, morphological and compositional analyses using atomic force microscopy (AFM), scanning electron microscopy (SEM), high‐resolution transmission electron microscopy (HRTEM), energy dispersive X‐ray analysis (EDX), and X‐ray photoelectron spectroscopy (XPS).

**Figure 3:**
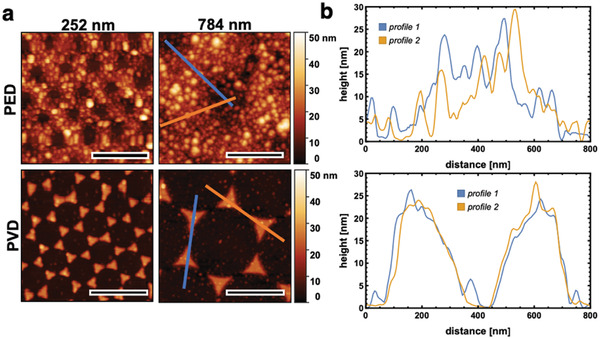
a) AFM scans of the PED and PVD photoelectrode surfaces using PS particle sizes of 784 and 252 nm, respectively. Colored lines in the 784 nm images highlight the cross‐sectional profiles shown in (b). b) Corresponding height profiles of PED (upper row) and PVD (bottom row) samples fabricated with 784 nm PS particle sizes. The scale bar in (a) is 500 nm.

AFM images of the PED samples (**Figure** **3**a) clearly show that the photoelectrodeposition of rhodium on the p‐InP surface results in a nanosized, 2D honeycomb structure.

**Figure 4:**
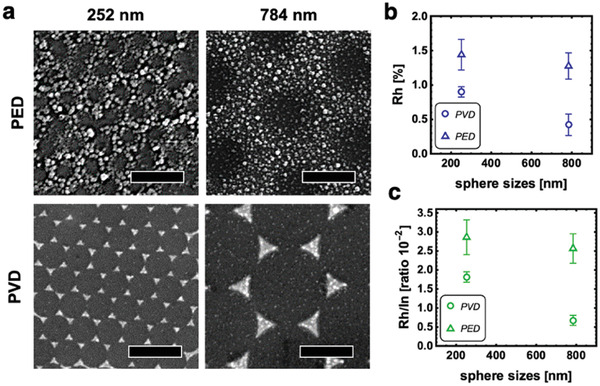
a) SEM images of the PED and PVD photoelectrode surfaces using PS particle sizes of 784 and 252 nm, respectively. The scale bar is 500 nm. b) Rh content [%] of the electrode surface determined in EDX survey scans at 50 000 magnification, each scanning an area of 8.2 µm × 5.5 µm. c) Rh/In ratio determined in EDX survey scans using the same parameters as in (b).

The hole diameter is determined by the PS particle size, whereas some Rh grains can also be found inside the holes. The Rh is grained coarsely, with single particle sizes between 10 and 32 nm for the 252 nm sample and between 15 and 33 nm for the 784 nm sample, respectively (Figure S4, Supporting Information). Interestingly, the smaller PS particle size results in a larger distribution of smaller and larger Rh grains, almost following a Gaussian distribution pattern centered around 18 nm. The 784 nm PED sample shows on the contrary a smaller distribution pattern of Rh grain sizes, with most grains being about 15–24 nm in size. The difference in Rh grain size and size distribution suggests that the PS particle size influences the Rh grain size upon photoelectrodeposition. Physical vapor deposition of the Rh results however in hexagonally aligned triangular structures with very fine Rh grains which are not distinguishable in the AFM images. Although the height profiles of the PED and PVD samples are similar with respect to the height of the catalytic ‘hot spots’ (about 30 nm, Figure 3b), the AFM images show that the surface morphologies are fundamentally different. The size of the photoelectrocatalytic “hot‐spots” is determined by the size of the PS sphere, whereas the Rh grain size is dependent on the deposition technique. SEM studies (**Figure** **4**a) confirmed the homogenous catalytic array of Rh ‘hot spots’ in the PED and PVD structures and electron beam diffraction and TEM investigations reveal the nanocrystalline cubic structure with an interplanar spacing of 2.2 Å for both, PED and PVD samples, as well as an 111 orientation (Figure S5, Supporting Information). XP spectra of the PED and PVD electrode surfaces (Figures S6 and S7, Supporting Information) show moreover that all four electrodes have a significant InOx/POx oxide layer on the electrode surface after the drop experiments. Generally, the InPO_4_ contribution is higher in PED samples, as shown in the P 2p (peak position: 133.4 eV) and the In 3d spectra (peak position: 445.6 eV), whereas the In(PO_3_)_3_ contribution is higher in the PVD samples (peaks at the binding energies 134.4 and 444.8 eV, respectively). The P 2p and In 3d spectra indicate as well that InP remains the dominant In compound on the electrode surface, whereas the PVD samples generally show a higher atomic percentage (60.64% in the 252 nm PVD sample and 43.01% in the respective PED sample and 44.72% in the 784 nm PVD sample and 32.53% in the respective PED sample). This can be explained by the different Rh electrocatalyst surface coverages of the PED and PVD samples which is already evident from the AFM images in Figure 3 and which will also be discussed in more detail below. The Rh is also partly oxidized in all samples after the drop tower experiments, with Rh_2_O_3_ (308.3 eV) and RhO_2_ (309.8 eV) being the dominant oxidized species. Control XP spectra with 784 nm PVD and PED samples prior to the drop tower experiments (Figure S7, Supporting Information) suggest that Rh oxidation occurred in both samples prior to the drop experiment, potentially during the pre‐treatment of electrodes before photoelectrochemical testing when the PS sphere mask was removed. A p‐InP oxide layer formed as well during the pre‐treatments which was removed before the drop tower experiments by potentiostatic cycling in 1 m HCl(aq). This is likely the reason for the higher InP amount present on the electrode surface before the drop experiment. During the drop, oxide layers form again, because the open areas of the PS‐sphere prepared structure are directly exposed to the electrolyte and show enhanced emission from InP covered by a thin layer of InPO_4_. The fact that the XPS spectra of the PED and PVD samples are overall not significantly different implies a very similar surface chemical environment for both types of electrodes. EDX analyses of the electrodes reveal however differences in the overall Rh content which can also be estimated from AFM and SEM images as discussed above (Figure 4b). The overall Rh content on the p‐InP surface is nearly 1.7 times higher on the 252 nm PED sample compared to the 252 nm PVD sample. The 784 nm PS particle size samples have slightly less Rh on the surface than the corresponding 252 nm samples. A calculation of the Rh atomic percentage (Figure 4b) and the Rh/In ratio (Figure 4c) support this trend: the PED samples show a higher percentage of Rh deposits on the surface (e.g., 1.5 ± 0.5% (PED) and 0.9 ± 0.1% (PVD), respectively, both 784 nm samples) and generally exhibit a higher Rh/In ratio on the surface than the PVD ones. This explains the initially better performances of the PED samples in microgravity environment—especially, the high current densities observed with the 252 nm PED samples. It implies that the initially larger number of available catalytic sites on the 252 nm PED sample is the main reason behind the high photocurrent densities at the beginning of the measurement. However, it does not explain the significant photocurrent density decrease observed in the CA and CV measurements during the course of the experiment. Along with the similar surface chemical environment elicited by XPS, the surface analysis results suggest that the structural and morphological differences play a significant role for the photoelectrocatalytic efficiency.

### 2.4 Hydrogen Gas Bubble Formation in Reduced Gravitation

To verify this hypothesis further, we firstly calculated (further details in the Supporting Information) the hydrogen gas evolution efficiency (*f*_G_) of the different electrodes based on our video recordings during the 9.2 s of free fall at the time points 2.3, 4.6, 6.9, and 9.2 s by calculating the volume ratio of produced hydrogen gas (*V*_G_) and the theoretically expected gas volume (*V*_T_) according to the Faraday equation

(2) $$f_{G}=\frac{V_{G}}{V_{T}}\;$$

A comparison between the PVD and PED samples reveals that the 252 nm PED sample shows very high *f*_G_ values during the course of the experiment (Figure S8, Supporting Information) which saturate at a value of 0.68 after 6.9 s and decreases slightly thereafter to 0.65 at 9.2 s at the end of the drop sequence. All other samples demonstrate steady *f*_G_ increases during the 9.2 s, which result in final values of 0.25 (784 nm PED sample), 0.59 (252 nm PVD sample), and 0.48 (784 nm PVD sample), respectively. This analysis suggests that it is indeed the surface morphology which is of key importance to a high solar‐to‐hydrogen efficiency in reduced gravitation: despite higher amounts of Rh deposits on the 252 nm samples (and therefore, more available catalytic sites)—the gas evolution efficiency analysis reveals that the 784 nm PVD sample exhibits the highest, continuous hydrogen production rate during free fall. This is also reflected in the earlier discussed photoelectrochemical measurements. To investigate the discrepancy between the steep increase and saturation of *f*_G_ of the 252 nm sample and the continuous *f*_G_ increase of the PVD samples, we investigated the electrode surface wettabilities. It is known from earlier water electrolysis experiments in microgravity environment that altering the electrode surface wettability, e.g., by introducing self‐assembled monolayers (SAMs) influences the overall cell characteristics significantly.^[^ ^16^ ^]^

**Figure 5:**
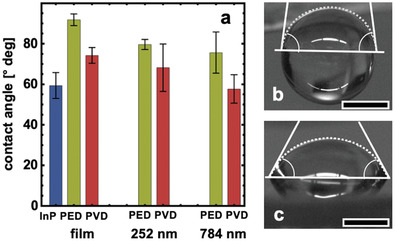
a) Statistical analysis of static contact angle measurements of an electrolyte droplet containing 1 m HClO_4_ with the addition of 1% (v/v) isopropanol on PVD and PED electrodes fabricated with the indicated PS particle sizes. Results from the bare p‐InP electrode and continuous Rh films formed by PED and PVD are shown as a comparison. b,c) Static droplets on 784 nm PEC sample and on a 784 nm PVD sample, respectively. The scale bar is 1 mm. All measurements were carried out under ambient and terrestrial conditions.

Static contact angle measurements with electrolyte droplets containing 1 m HClO_4_(aq) with the addition of 1 (v/v)% isopropanol were used to determine the gas bubble contact angle and moreover, the wettability of the electrode surface (**Figure** **5**).

Interestingly, both Rh deposition techniques, PED and PVD, result in a more hydrophobic electrode surface compared to the bare p‐InP. The largest contact angle of about 92 ± 3° and therefore, the most hydrophobic sample—is observed when a continuous Rh film is photoelectrodeposited on the electrode surface. The PVD structures exhibit significantly lower contact angles than the rougher PED structures (Figure 5b,c), with the 784 nm PVD sample showing nearly the same surface hydrophilicity as the bare p‐InP electrode (contact angle about 58 ± 3°). This result is crucial for the interpretation of the photoelectrochemical experiments in Figure 1, as the gas bubble desorption diameter depends directly on the gravitational force and the gas bubble contact angle as summarized by the Fritz equation

(3) $$d_{0}=\;1.2\;\theta \sqrt{\frac{\gamma }{g\;\left(\rho_{L}-\rho_{G}\right)}}$$

Here, the gas bubble diameter upon desorption from the electrode surface is *d*_0_, the gas bubble contact angle is *θ* (in degrees), surface tension is *γ*, gravity is *g*, and the densities of surrounding liquid and the respective gas are *ρ*_L_ and *ρ*_G_, respectively.^[^ ^36^ ^]^ Strictly speaking, the Fritz equation is only valid at zero current and the gas bubble break‐off diameter *d* has been experimentally found to be correlated to *d*_0_^[^ ^36^, ^37^ ^]^

(4) $$d\;=\frac{d_{0}}{1+50\Theta }\;$$

whereas Θ is the fractional gas coverage expressed by

(5) $$\Theta =\;0.023{\left(\frac{J}{A}\right)}^{0.3}$$

**Figure 6:**
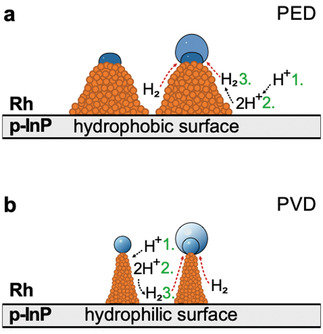
Cross‐sectional scheme of hydrogen gas bubble evolution on the a) PED and b) PVD nanostructured photoelectrodes, illustrating the difference in the electrode surface hydrophilicity and the respective gas bubble formation process.

Here, *J* is the photocurrent density and *A* is the electrode surface area. It is evident that in microgravity environment (10^−6^ *g*), the radicand in Equation (2) becomes very large. This emphasizes the importance of a high electrode surface hydrophilicity and a small gas bubble contact angle for enhanced gas bubble desorption and an efficient photoelectrode operation in reduced gravitational environment: the pyramidal structure of the PVD samples result in lower gas bubble contact angles which lead to smaller gas bubble detachment diameters according to Equation (2). The correlation is also in very good agreement with the observation made for the 784 nm PVD sample, which demonstrated the highest, most stable photocurrent densities during the 9.2 s of free fall. The gas evolution efficiency data in Figure S8 (Supporting Information) furthermore suggest that continuous gas bubble release occurred from the electrode surface. The smaller gas bubble contact angle translates to weaker frictional forces at the small gas/liquid interface which cause enhanced gas bubble detachment from the electrode surface.^[^ ^16^ ^]^ Despite the higher activity of the 252 nm PED sample due to the larger amounts of Rh present on the electrode surface, the photoelectrode efficiency in microgravity drops over time due to the gas bubble desorption from the electrode surface being hindered by the stronger interaction of gas bubble and electrode surface as demonstrated in surface wettability studies. The overall hydrogen evolution mechanism on both electrodes is relatively similar and depictured in **Figure** **6**. The introduced catalytic nanostructures form catalytic ‘hot‐spots’with locally enhanced electric fields at the tip^[^ ^38^ ^]^ which control the location of gas bubble formation and thus prevent initial coalescence. The produced gas is dissolved and accumulated in vicinity of the tips to form a supersaturation layer. Sakuma et al. have shown that the electrode surface wettability also influences the surface energy used to form the heterogeneous gas/solid interface which moreover affects the degree of supersaturation required before gas bubble nucleation.^[^ ^16^ ^]^

Under terrestrial conditions, the density gradient between supersaturation and bulk layers induces single‐phase free convection which is responsible for electrolyte mixing and the reduction of supersolubility in the vicinity of the electrode surface. In microgravity, the gas–liquid interface free energy predominantly controls bubble nucleation. The absorption rate of the dissolved gas through the gas/liquid interface is given by the gas evolution efficiency, *f*_G_, as defined above in Equation (1). Dissolved gas is absorbed more quickly on a hydrophobic electrode, resulting initially in higher *f*_G_ values which is also observed here. On more hydrophilic electrodes, the bubbles maintain a spherical shape and grow toward the bulk of the electrolyte with a lower concentration, resulting in poorer collection rates of dissolved gas. Due to the absence of buoyancy, gas bubble growth on the electrode surface is diffusion‐controlled and dissolved gas around the nucleation site diffuses toward the bubble. The hydrophilic electrodes produce smaller bubbles which can easily detach due to weaker frictional forces at the smaller gas/liquid interface preventing the bubbles from staying attached to the surface. The easier mobility also leads to moderate convective flows which enhance mass transport and ultimately leads to a higher amount of available electrocatalytic active sites.

Hindered or slow gas bubble detachment has been demonstrated to affect the activation and ohmic overpotentials in electrolysis due to decreasing the effective electrode surface area and blocking ion pathways available for current transport, whereas it could decrease concentration overpotentials by absorbing dissolved gas and thus decreasing supersaturation levels in the electrolyte. As discussed above, in photoelectrochemical systems, bubbles are furthermore prone to induce light scattering which affect the light absorption properties of the system. Given the absence of density‐driven convection processes, microgravity is an ideal environment to investigate the microconvectional forces on gas bubble growth and detachment in (photo‐)electrocatalytic systems and thus to optimize microconvectional processes such as mass transfer for an efficient device operation.

## 3 Conclusion

We investigated hydrogen evolution on p‐InP photoelectrodes coated with a nanostructured Rh layer fabricated by shadow nanosphere lithography in microgravity environment generated for 9.2 s at the Bremen Drop Tower. By depositing Rh via physical vapor deposition or photoelectrodeposition through polystyrene particle spheres sized 252 or 784 nm, respectively, we could judiciously control the wettability of the electrode surface and therefore, the hydrogen gas bubble detachment from the electrode surface even in the absence of buoyancy. Our results suggest that for efficient operation of (photo‐)electrochemical devices in terrestrial and reduced gravitational environments, hydrophilic surfaces are most efficient for gas bubble detachment due to the facilitation of small gas bubble contact angles which leads to spherical bubble growth. We have shown that the total photoelectrochemical device efficiency in reduced gravitational environment is an interplay between the optimal electrocatalyst amount on the electrode surface—providing sufficient catalytic sites for high rates of product formation—and the endurance of efficient gas bubble release through the spacing of gas bubble nucleation sites to prevent bubble coalescence as well as the creation of a hydrophilic surface with small gas bubble contact angles. Shadow nanosphere lithography has proven to be a successful technique to balance the above requirements for the design of efficient electrocatalyst nanostructures applicable in terrestrial and reduced gravitational environments without the need for additional surface chemical functionalizations which may impact the catalytic performance.

## 4 Experimental Section

### Preparation of the p‐InP Photoelectrodes

One‐sided, polished, single crystal (111 A orientation) p‐InP was obtained from AXT Inc. (Geo Semiconductor Ltd., Switzerland) with a Zn doping concentration of 5 × 10^17^ cm^−3^. Ohmic back contacts were fabricated by evaporation of 4 nm Au, 80 nm Zn, and 150 nm Au and consecutive annealing for 60 s at 400 °C. The wafer was then cut into 1 cm^2^ squares. Subsequently, the substrates were etched for 30 s in a freshly mixed bromine (0.05% (w/v))—methanol solution, rinsed with ethanol and ultrapure water, and dried under nitrogen flux. All solutions were made from ultrapure water and analytical grade chemicals with an organic impurity level below 50 ppb.

Conditioning of p‐InP was realized photoelectrochemically in 0.5 m HCl by voltammographic cycling between −0.44 and +0.31 V (*vs* RHE) at a scan rate of 50 mV s^−1^ in a three‐electrode setup while exposing the electrode surface to white light (100 mW cm^−2^) and purging with nitrogen gas of 5.0 purity. All photoelectric measurements including cyclic voltammetry and chronoamperometric measurements were performed in a standard three‐electrode potentiostat arrangement where a platinum‐coil electrode was used as counter electrode and an Ag/AgCl (3 m) was used as reference electrode. White‐light illumination was provided by a tungsten halogen lamp (Edmund Optics) and was shone through a quartz glass window into a borosilicate glass cell. A calibrated silicon reference electrode was used to adjust the light intensity before all experiments.

Rhodium nanostructure fabrication was carried out using SNL. Monodispersed beads of PS particles sized 784 nm and 252 nm were obtained at a concentration of 5% (w/v) from Microparticles GmbH and were further diluted. For the final solution of 600 µL, 300 µL of the PS‐beads dispersion was mixed with 300 µL ethanol containing 1% (w/v) styrene and 0.1% (v/v) sulfuric acid. The periodically aligned mask was created by applying the solution onto the air‐water interface using a Pasteur pipette with a curved tip. Gradual colored domains indicate crystalline ordered PS spheres. Gentle tilts of the petri dish allowed to increase the area of the monocrystalline domains by combining multiple smaller domains into larger ones. The solution was carefully distributed to cover about 80% of the water surface with a hcp monolayer, while leaving space for stress relaxation and avoiding formation of cracks in the lattice during the next preparation steps. Afterwards, the photoelectrochemically conditioned p‐InP electrodes were placed under the floating closed‐packed PS sphere mask in the Petri dish and the residual water was gently removed by pumping and evaporation until the mask deposited onto the electrode surface. The electrode surface was gently dried with N_2_(g) thereafter. Rhodium was either photoelectrochemically deposited through the PS spheres as described above to create honeycomb like nanostructures or deposited by electron beam evaporation to create periodically aligned triangular structures. Photoelectrochemical Rh deposition was carried out through the PS sphere mask in an electrolyte solution containing 5 ×10^−3^ m RhCl_3_, 0.5 m NaCl, and 0.5% (v/v) 2‐propanol at a constant potential of *V*_dep_ = +0.01 V *vs* RHE for 5 s under simultaneous illumination with a W‐I lamp (100 mW cm^−2^). The electrochemical specifications such as the electrochemical cell, reference, and counter electrode are the same as for the photoelectrochemical conditioning procedure. The photoelectrode was rinsed once with ultrapure water and dried under a gentle N_2_(g) flow. E‐beam evaporation was carried out in a customized high vacuum deposition chamber (VCH ≈ 0.1 m^3^) pumped by a typical dry turbo molecular pumping set with typical base pressure of 2 × 10^−7^ mbar. Deposition rates of 1.2 A s^−1^ for rhodium (≈1 kW e‐beam power) were used and controlled during deposition by quartz crystal microbalance to around 30 nm thickness. The Telemark evaporator operates with a 4 × 7 cm^3^ pocket source indexer. After the respective rhodium deposition process, the PS sphere mask was removed from the surface by placing the electrodes for 20 min under gentle stirring in a beaker with toluene. The sample was subsequently cleaned by rinsing with acetone, ethanol, and ultrapure water for 20 s each. To remove residual carbon from the surface, argon plasma cleaning was used for 6 min at a process pressure of 0.16 mbar, 65 W, and a gas inflow of 2 sccm.

### Photoelectrode Surface Characterization

Tapping mode atomic force microscopy (TM‐AFM) was used for the characterization of the surface morphology using a JPK NanoWizzard Ultra AFM and Oxford Instruments AC170TS probes with a radius of 7 nm.

SEM microscopy work was carried out using a Thermo Scientific Apreo SEM, with a high stability Schottky field emission gun, a Trinity Detection System, and a UltraDry EDX spectroscopy attachment.^[^^39]^ Surface EDX scans analyze an area of 8.2 µm × 5.5 µm, at a working distance of 10 mm, acceleration voltage of 10 kV, and a beam current of 40 nA.

XPS analysis was carried out for all samples. Samples were stored in nitrogen atmosphere prior to analysis. XPS analyses were performed in an ultrahigh‐vacuum instrument, with a base pressure below 5 × 10^−9^ mbar, with an aluminum X‐ray source (1486.6 eV, 300 W, XR 50, Specs GmbH, Berlin, Germany) and a hemispherical electron analyzer (Phoibos 100, Specs GmbH, Berlin, Germany). The analyzer has an iris diameter of 60 mm and an entrance slit size of 7 mm. Emission angle normal (90°) to the surface was used. A pass energy of 100 eV was used for all survey scans and 30 eV for all regional spectra collection. The alkyl carbon peak (C—C, C—H) at 284.8 eV was used as an internal reference for the binding energy scale. Backgrounds were generated with the Shirley method if the intensity of the spectrum was higher at higher binding energies; if not, linear backgrounds were used.^[^ ^40^, ^43^ ^]^ Detailed analysis of regional peaks was conducted using CasaXPS software. Most peak fittings used 70% Gaussian and 30% Lorentzian (GL(30) setting) peaks, except for metallic species (Rh and In) and indium oxide component peaks for which Doniach Sunjic functions were used. Indium peaks were fitted using the same methods by Muñoz et al.^[^ ^33^, ^42^, ^43^, ^44^, ^45^, ^46^ ^]^

Samples for transmission electron microscopy were prepared in a nitrogen filled glovebox (maximum pressure 0.2 ppm). A drop of the particle's solution in alcohol (after scratching off the surface of the substrate) was placed onto a carbon‐coated copper mesh grid and allowed to dry. The grid was then transferred in an air‐free container into the Phillips CM12 microscope, which was equipped with a 9800 EDAX analyzer. Several grids were prepared from each substrate to ensure that the procedure yielded reproducible samples for analysis. The samples were characterized in the in high‐resolution transmission electron microscopes, operating at 120 kV. The quality of the lattice images was improved using the conditions of minimum phase contrast according to Kunath et al.^[^ ^47^ ^]^

### Photoelectrochemical Experiments in Microgravity Environment

Microgravity environment was realized at the Bremen Drop Tower at the Centre of Applied Space Technology and Microgravity (ZARM), Germany. The experiment was installed in a drop capsule which was shot up by hydraulically controlled pneumatic piston‐cylinder catapult system about 120 m to the top of the tower before falling down again into a deceleration container containing millimeter‐sized hard foam polystyrene beads. The total free fall time in which microgravity is generated can be up to 9.3 s. During free fall, the minimum *g*‐value approached was about 10^−6^ *g*. Photoelectrochemical experiments in the drop tower were carried out in a custom‐made, two‐compartment photoelectrochemical cell (filling volume of each compartment: 250 mL) made of polyether ether ketone (PEEK). Each cell consisted of two optical windows made of quartz glass (diameter: 16 mm) through which front and side of the working electrode surface could be observed via optical mirrors and beam splitters. The front optical window was also used for illumination of the electrode surface with white light. Each cell could perform one photoelectrochemical measurements independently of the other cell. The experiments were carried out in a three‐ electrode arrangement with a Pt coil counter electrode and an Ag/AgCl (3 m) reference electrode. 1 m HClO_4_(aq) with the addition of 1% (v/v) isopropanol was used as an electrolyte to reduce the surface tension and initially favor gas bubble release. XPS and terrestrial, photoelectrochemical control experiments did not show any effect of the isopropanol on the chemical characteristics of the photoelectrodes. Light intensities of 50 and 100 mW cm^−2^ were provided by a W‐I white‐light source (Edmund Optics). All experiments were carried out under ambient pressure in the drop capsule.

Two cameras (Basler AG; acA2040‐25gc and acA1300‐60gm NIR, lens types: 35 mm Kowa LM35HC 1″ Sensor F1.4 C‐mount and Telecentric High Resolution Type WD110 series Type MML1‐HR110, respectively) were attached to each cell via optical mirrors (monochromatic camera, side) and beam splitters (color camera, front) to reduce the momentum on the camera setup by the hard acceleration processes allowing to record a static view of the gas bubble formation in microgravity conditions. Data were stored during each drop on a Matrox 4Sight GPm integrated PC unit in the drop capsule. Single pictures were recorded at a frame rate of 25 fps (front camera) and 50 fps (side camera). Electrical power in the capsule was provided by batteries. Drop sequences for cyclic voltammetry and chronoamperometric measurements were automated and started prior to each drop waiting for triggers at launch. The drop sequence was designed to start cameras, illumination sources and potentiostat measurements while simultaneously immersing the working electrode into the electrolyte using a pneumatic system in time reaching microgravity conditions. This allowed photoelectrochemical measurements such as cyclic voltammetry and chronoamperometric measurements to be carried out only during the 9.2 s of microgravity. It is important to immerse the photoelectrode only in the electrolyte during the experimental time in microgravity to prevent morphological and chemical changes before and after the experiments. After the capsule was lifted from the deceleration container, the samples were retrieved from the experimental setup, rinsed with MilliQ water and dried with pure N_2_(g) flow before storage in N_2_(g) atmosphere until further investigations were carried out.

### Terrestrial IMVS, IMPS, and IPCE Measurements

IPCE measurements were performed with a 300 W Xe lamp (LOT‐Oriel) coupled with a grating monochromator (Acton Spectra Pro 2155) and long‐pass filters (3 mm thick, Schott) to remove higher order diffraction. A Si photodiode (Hamamatsu, S1337‐BQ) was used to measure the intensity of the monochromatic light. The IPCE values were calculated using the following equation

(6) $$\mathrm{IPCE}\;\left(\%\right)=\frac{j_{\mathrm{ph}}\left(\lambda \right)}{P_{\mathrm{mono}}\left(\lambda \right)}\;\cdot \frac{1240}{\lambda }\cdot 100$$

Here, *j*_ph_(*λ*) is the measured photocurrent (in mA cm^−2^), *P*(*λ*) is the calibrated and monochromated illumination power density (in mW cm^−2^) at each wavelength *λ* (in nm) and 1240 V nm represents the multiplication of *h* (Planck's constant) and *c* (speed of light).

IMVS and IMPS measurements were performed in the same three‐electrode configuration and cell used for the other PEC experiments. A Solartron 1286 potentiostat was used to measure the open‐circuit voltage during IMVS measurements and control the applied potential during IMPS measurements. Modulated illumination was provided by a 455 nm wavelength LED (Thorlabs M455L3), which is driven by an LED driver (Thorlabs DC2100). A frequency response analyzer (FRA, Solartron 1255) was used to provide the sinusoidal modulation (frequency range 1 Hz to 100 kHz) of the LED illumination (rms amplitude of 0.48 mW cm^−2^ superimposed on 4.33 mW cm^−2^ DC background intensity) as well as recording the real and imaginary value of the photovoltage (IMVS) and photocurrent (IMPS). The charge recombination rate constants, *k*_rec_, and the charge transfer rate constants, *k*_tr_, in IMPS measurements were calculated from low‐ and high‐frequency intercepts (LFI and HFI) as well as *f*_max_ of the recombination circle according to LFI/HFI = *k*_tr_/(*k*_tr_ + *k*_rec_).

The spectrum of the W‐I lamp (Edmund optics MI – I150 with Ushio EKE/L 21 V 150 W lamps) used for the setup, including the 50 cm glass light guides, was measured using a calibrated CCD spectrometer (USB2000+, Ocean Optics). Wavelengths <350 nm are found to be absorbed by the light guides, whereas the optical band gap of p‐InP (1.34 eV, equivalent to 925 nm) represents the upper limit of the absorption spectrum. Equation (6) was used to calculate the characteristic spectra for each nanostructured photoelectrode using the W‐I light source which was used in the microgravity experiments (100 mW cm^−2^) and a reference AM 1.5 global tilt solar spectrum.^[^ ^48^ ^]^ To compare the photocurrent densities generated by the different nanostructured p‐InP photoelectrodes without accounting for changes to the absorption spectrum through external parameters such as different gas bubble desorption characteristics in terrestrial and microgravity environments, the spectra were normalized with respect to the 784 nm PVD samples.

### Theoretical Simulations

Theoretical simulations of the current–voltage behavior of the PEC cell were carried out as described previously.^[^ ^18^ ^]^ The following assumptions based on experimental observations have been made. The catalytic exchange current density of Rh, *j*_0,cat_ was assumed to stay the same in microgravity environments and was set to *j*_0,cat_ = 0.1 mA cm^−2^, which is consistent with experimental reports in the literature for Rh as a hydrogen evolution catalyst.^[^ ^18^ ^]^ For the InP|Rh Schottky junction, the dark current (*j*_0_) is assumed to be 10^−8^ mA cm^−2^. Due to the InP*_x_* O*_y_* layer, the ideal equations for the dark current of a Schottky junction did not accurately describe the system. Therefore, this value is based on a fit to the experimentally measured current–voltage curves. The *f*_SA_ value for the nanostructured 784 nm PED photoelectrode is 1.1 which is based on the surface area of the catalyst as determined from AFM data as used previously. The light‐limited photocurrent density *j*_L_ was set to 35 mA cm^−2^ which was also used in previous calculations.

## Conflict of Interest

The authors declare no conflict of interest.

## Supporting information

Supporting Information

Supplemental Video

Supplemental Video

Supplemental Video

Supplemental Video

Supplemental Video

Supplemental Video

Supplemental Video

## Data Availability

The data supporting the findings of this study are available from the corresponding author upon reasonable request.
