# Supplementary material for: Releasing the Bubbles: Nanotopographical Electrocatalyst Design for Efficient Photoelectrochemical Hydrogen Production in Microgravity Environment
Source: Adv Sci (Weinh). 2022 Jan 21;9(8):2105380. doi: 10.1002/advs.202105380 (PMC8922132; doi:10.1002/advs.202105380)
Supplement: Supplementary file 1 — Supporting Information [file ADVS-9-2105380-s004.pdf]

ADVANCED SCIENCE
Open Access

Supporting Information

for *Adv. Sci.*, DOI 10.1002/advs.202105380

Releasing the Bubbles: Nanotopographical Electrocatalyst Design for Efficient Photoelectrochemical Hydrogen Production in Microgravity Environment

Ömer Akay, Jeffrey Poon, Craig Robertson, Fatwa Firdaus Abdi, Beatriz Roldan Cuenya, Michael Giersig and Katharina Brinkert**

ADVANCED SCIENCE
Open Access

Supporting Information

for *Adv. Sci.*, DOI: 10.1002/advs.202105380

Releasing the Bubbles: Nanotopographical Electrocatalyst Design for Efficient Photoelectrochemical Hydrogen Production in Microgravity Environment

Ömer Akay, Jeffrey Poon, Craig Robertson, Fatwa Firdaus Abdi, Beatriz Roldán Cuenya, Michael Giersig, Katharina Brinkert**

Supporting Information

Releasing the Bubbles: Nanotopographical Electrocatalyst Design for Efficient Photoelectrochemical Hydrogen Production in Microgravity Environment

Ömer Akay, Jeffrey Poon, Craig Robertson, Fatwa Firdaus Abdi, Beatriz Roldán Cuenya, Michael Giersig, Katharina Brinkert**

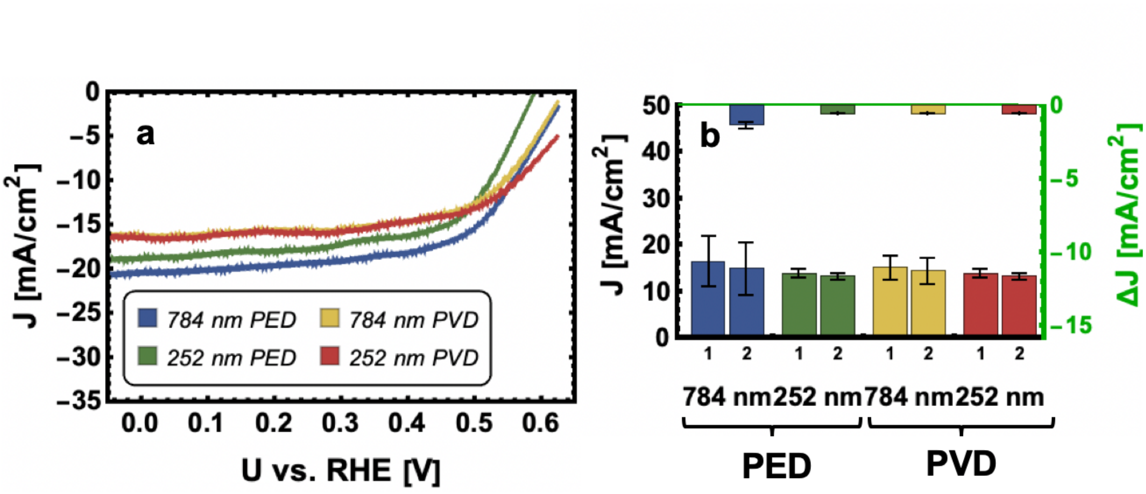

Figure SI 1 *a) Cyclic voltammograms of p-InP photoelectrodes coated with nanostructured Rh electrocatalyst layers recorded in an electrolyte containing 1M $HClO_4$(aq) with the addition of 1% (v/v) 2-propanol and a light intensity of 50 mW cm^{-2} (W-I lamp) during 9.2 s of free fall. The second reduction cycles out of three fully recorded cycles are shown. The scan rate was 326 mV s^{-1}. b) The photocurrent density difference between the first and second reduction cycle was determined statistically. The total current density drop is shown on the second y-axis. The inset in a) indicates the used PS particle size and Rh deposition technique.*

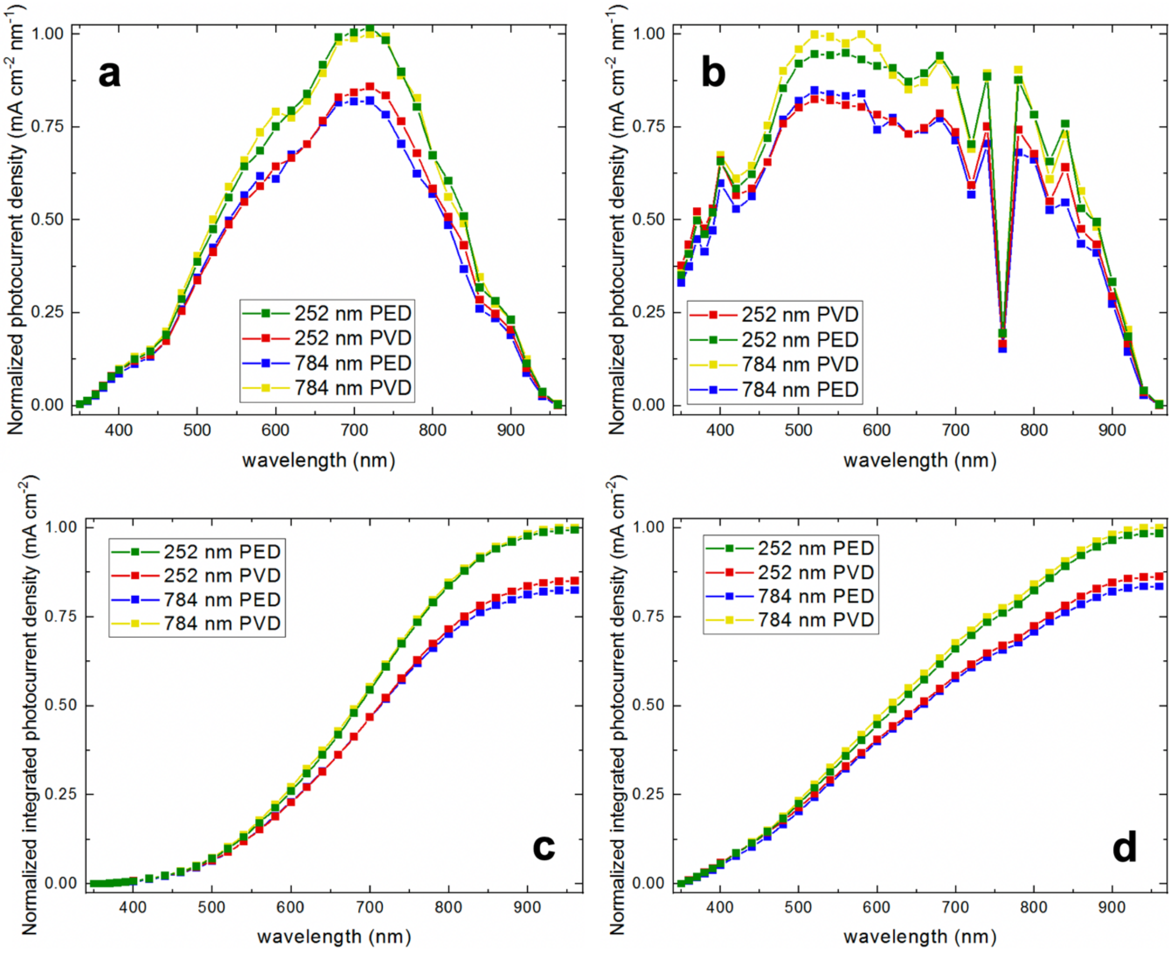

***Figure SI 2** Normalized photocurrent densities per wavelength obtained with p-InP photoelectrodes coated with nanostructured Rh electrocatalyst layers recorded in an electrolyte containing 1M $HClO_4$(aq) with the addition of 1% (v/v) 2-propanol. The photocurrent densities of the different samples were normalized to the best performing photoelectrode, 784 nm PVD, to account for external factors influencing the photocurrent generation in microgravity and terrestrial environments such as differences in the gas bubble desorption behaviours. (a) Normalized photocurrent densities obtained using a W-I lamp with a light intensity of 100 mW cm^{-2}, (b) normalized photocurrent densities obtained using a Xe lamp (AM 1.5 G), (c) normalized integrated photocurrent densities obtained with a W-I lamp (100 mW cm-2 illumination) and (d) normalized integrated photocurrent densities obtained with a Xe lamp (AM 1.5 G illumination).*

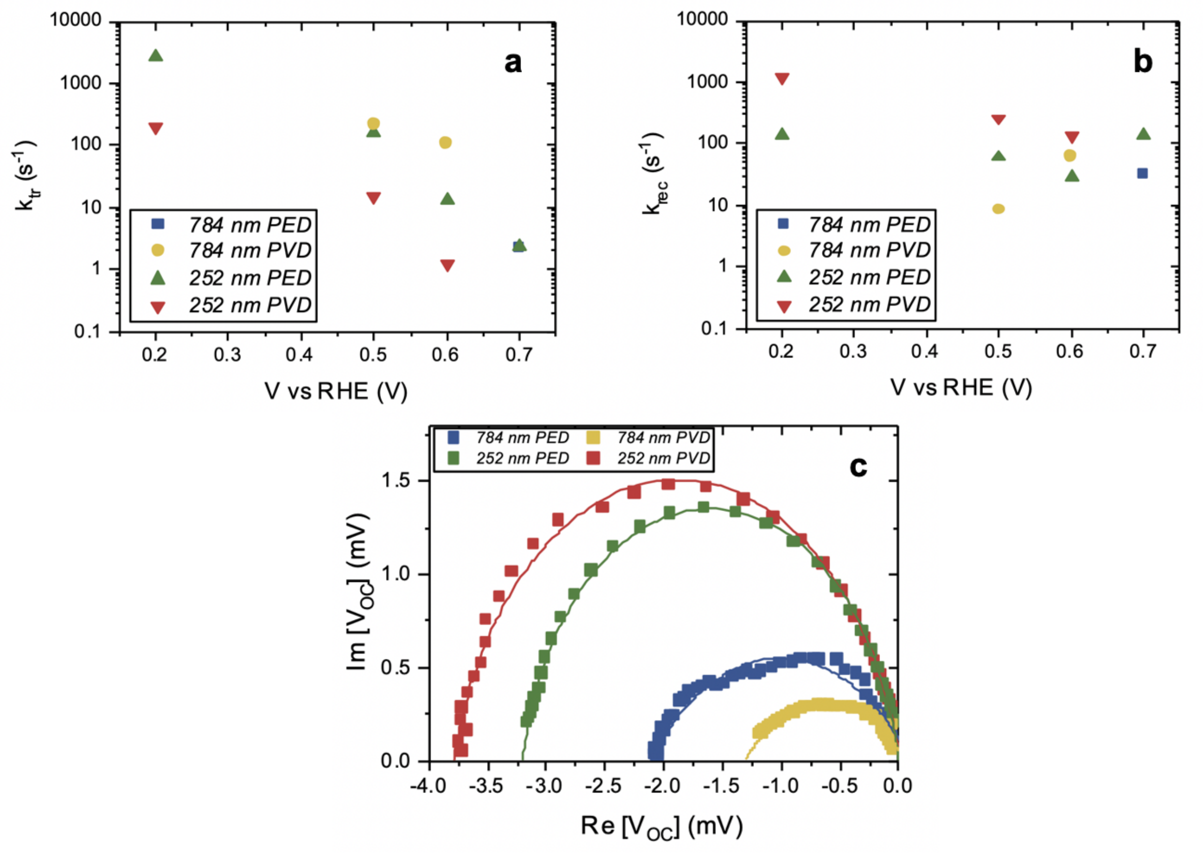

Figure SI 3 *Charge transfer (k_{tr}, (a)) and recombination rate constants (k_{rec} (b)) and extracted from terrestrially obtained IMPS data at different applied potentials in and electrolyte of 1 M $HCLO_4$(aq) with the addition of 1% (v/v) 2-propanol for the different p-InP-Rh photoelectrodes utilised in microgravity experiments under monochromic illumination (λ = 455 nm, see Experimental Section for details). Only data where recombination semicircle were present in the Nyquist plot were analyzed to calculate the shown rate constants according to $k_{tr} + k_{rec} = 2\pi f_{max}$. (c) Nyquist plot of IMVS measurements of the p-InP-Rh photoelectrodes utilized in microgravity environment using the same electrolyte and illumination specifications as in (a) and (b). Electron lifetimes are calculated according to $\tau_n = (2\pi f_{max})^{-1}$.*

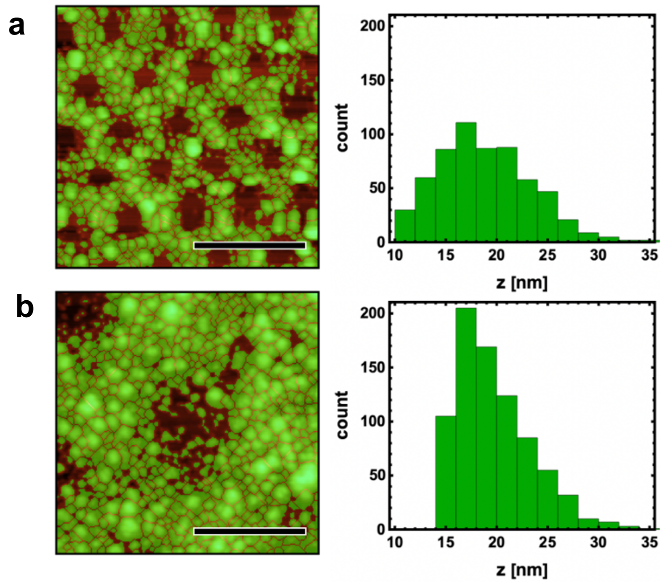

Figure SI 4 *AFM scans of the PED photoelectrode surfaces using PS particle sizes of 252 nm (a) and 784 nm (b), respectively. The different grain sizes of the Rh resulting from the photoelectrodeposition are highlighted in green colours and are shown again on the right according to their occurance on the electrode surface. The scale bars in (a) and (b) are 500 nm, respectively.*

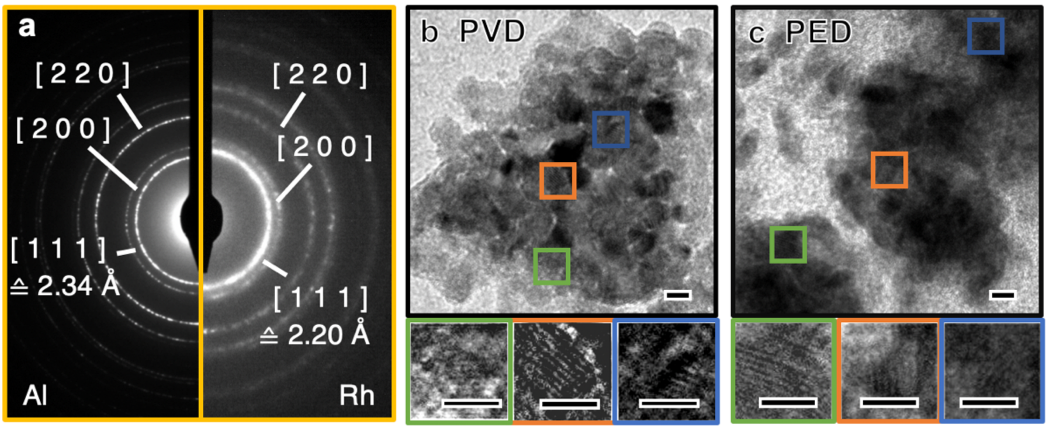

Figure SI 5 *a) Electron diffraction pattern of the Rh electrocatalyst (right). Al-foil is used for calibration (left). The lattice plane spacing is 2.2 Å which is typical for cubic structures. b) HRTEM images of Rh from single hotspots formed via PVD and c) PED at different magnifications. The scale bars indicate a resolution of 4 nm.*

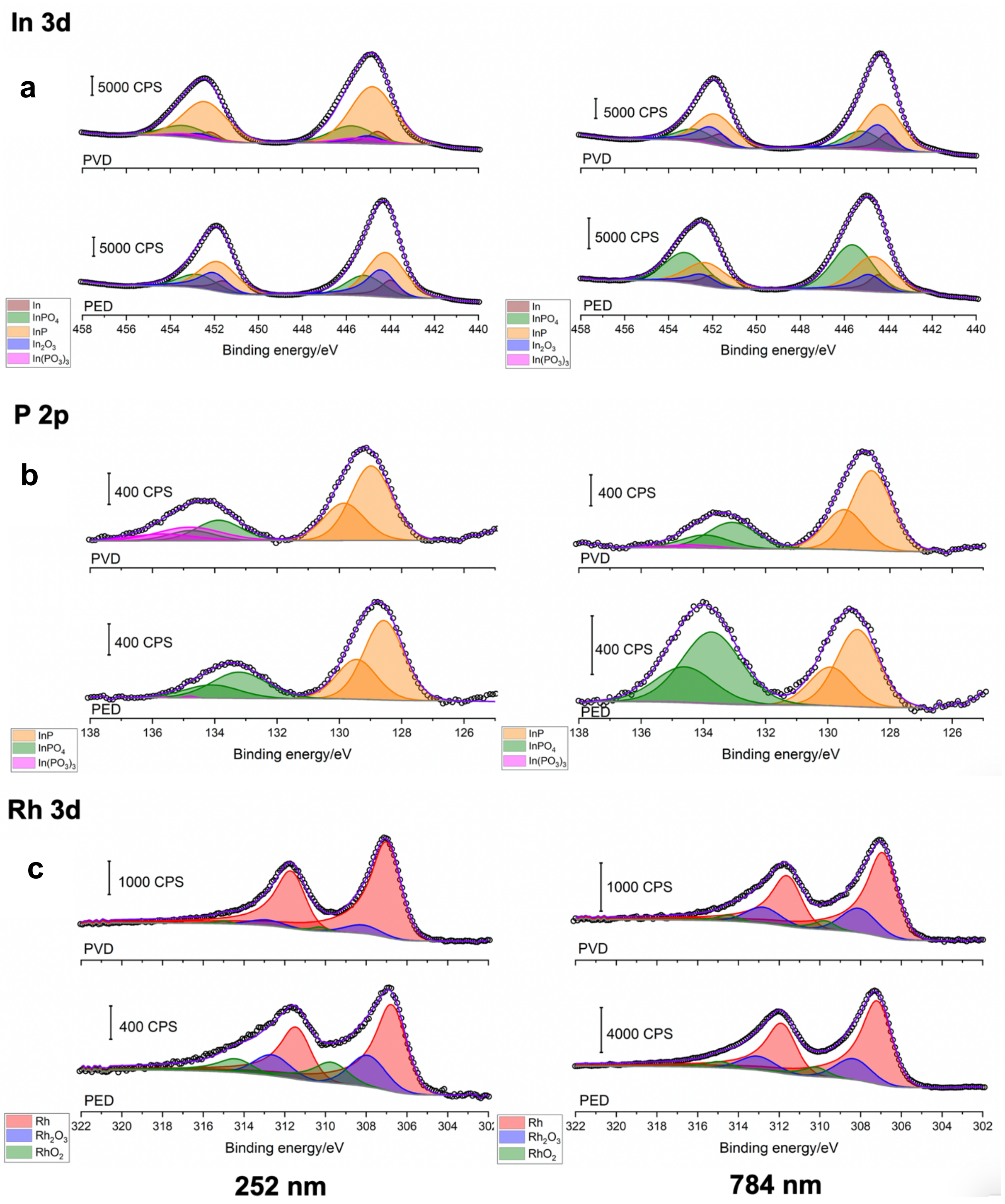

Figure SI 6 *X-ray photoelectron spectra of the p-InP/Rh photoelectrodes after the drop experiment with Rh nanostructures fabrictated using 252 nm (left) or 784 nm (right) PS particle sizes and PVD or PED as Rh deposition techniques, respectively. Shown are the In 3d core levels (**a**), the P 2p core levels (**b**) and the Rh 3d core levels (**c**). The colour coding under the lines prefers to the respective composition as shown in the legend.*

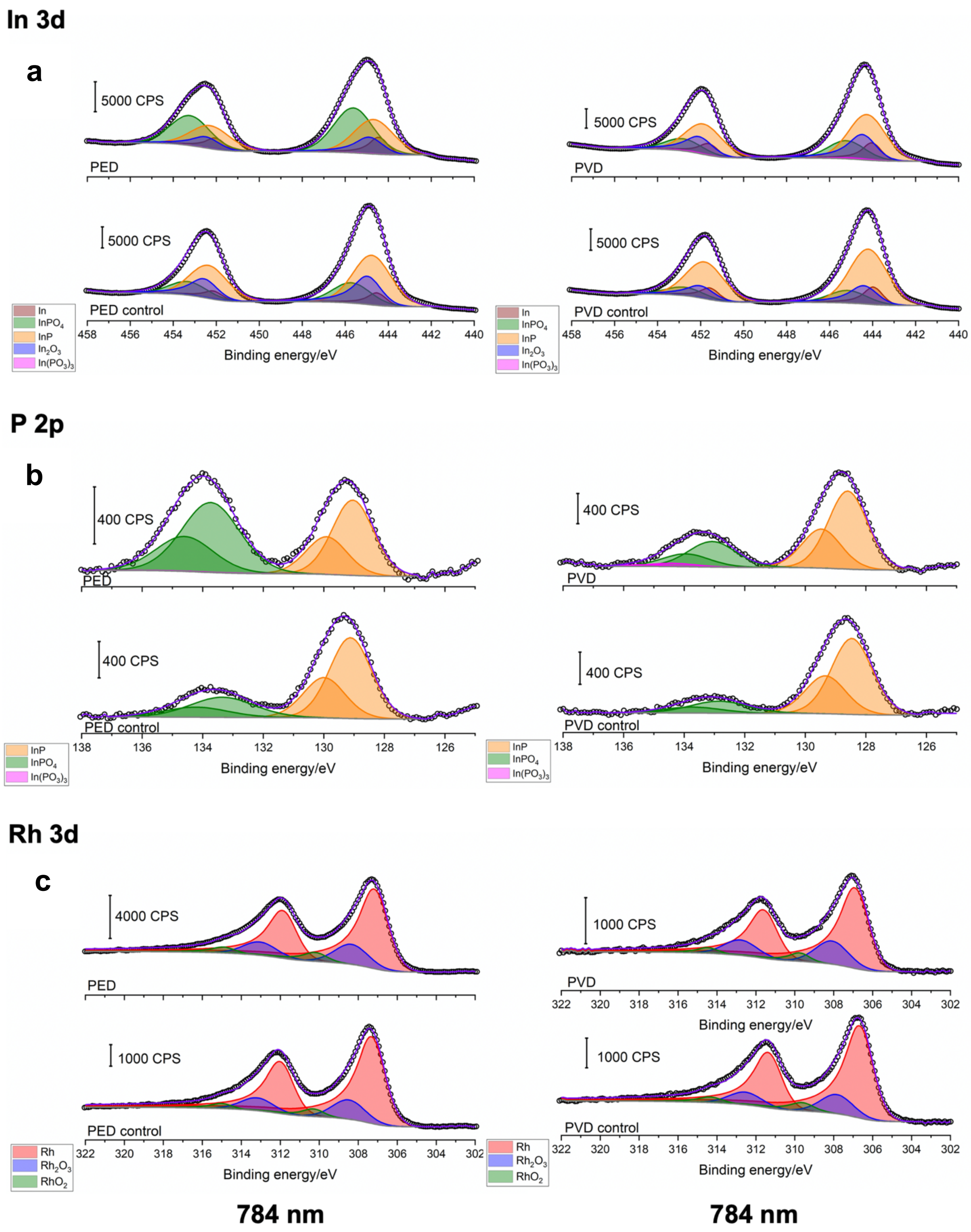

Figure SI 7 *X-ray photoelectron spectra of the p-InP/Rh photoelectrodes with Rh nanostructures fabrictated using 784 nm PS particle sizes and PED (left column) or PVD (right column) as Rh deposition techniques, respectively. Shown are the In 3d core levels (**a**), the P 2p core levels (**b**) and and Rh 3d core levels (**c**). The colour coding under the lines prefers to*

*the respective composition as shown in the legend. The first, third and fifth row in (**a**)-(**c**) are spectra recorded from samples after the drop experiment and the second, forth and sixth row in (**a**)-(**c**) are control spectra recorded directly after electrode fabrication and before the drop tower experiment.*

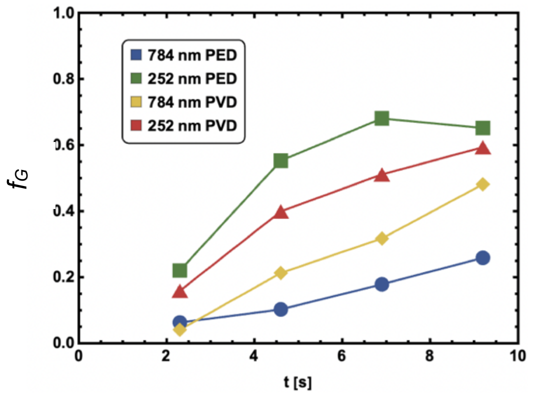

Figure SI 8 *Hydrogen gas bubble evolution efficiencies calculated for the duration of the drop experiment (9.2 s) at the time points 2.3 s, 4.6 s, 6.9 s and 9.2 s for the PED and PVD photoelectrodes with PS particle sizes of 252 nm or 784 nm.* f_G *is calculated from the ratio of produced hydrogen gas (*V_G*) as determined from recorded videos during the drop and the theoretically produced gas volume (*V_T*) calculated according to the Faraday equation.*

Supplementary Note 1.

Calculation of the gas evolution efficiency

In order to calculate the gas evolution efficiency of the photoelectrodes in reduced gravitation, the gas bubble surface coverage was firstly calculated using the recorded videos during free fall. Single images at 2.3 s, 4.6 s, 6.9 s and 9.2 s were selected, representing a quarter, half, three-quarter and final marks of the experiment. The catalytically active electrode area (A_s) and the area which was covered with hydrogen gas bubbles (A_b) was determined using the open access software *ImageJ*, from which the total electrode surface coverage (θ) could be calculated:

$$\theta = \frac{A_b}{A_s} \cdot 100 \qquad (1)$$

The area covered by gas bubbles on the surface was divided by the mean size of a bubble at the specific time periods for estimating the number of bubbles at the respective time points. The mean diameter was calculated from side camera images. The volume of gas bubbles on the surface was then calculated using the following equation:

$$V_s = n \cdot \frac{\pi d^3}{6} \qquad (2)$$

Where V_S is the volume of gas bubbles on the surface, n is the approximate number of gas bubbles and d is the mean diameter of a bubble at that time period. By adding this volume to the sum of the volume of bubbles that have left the surface before a certain time period, the

total volume of hydrogen, V, that had been produced by the system prior to a certain time point could be calculated.

Based on the current density data, the expected, theoretical volume of hydrogen could be calculated according to the following equation:

$$V_x = \frac{V_m\, I\, t}{n\, F} \qquad (3)$$

Where V_X is the expected volume, V_m is the molar volume (24465403.7 mm^3 mol^{-1} at 25 °C), I is the current in Amperes (A), t is the time elapsed in the experiment to that point in seconds (s), n is the number of transferred electrons in the reaction and F is Faraday's constant. Using both, the theoretically expected volume and the calculated, actual volume produced, the gas evolution efficiency, f_G, can be calculated according to the following equation:

$$f_G = \frac{V}{V_x} \qquad (4)$$
